# MicroRNA-21a-5p-modified macrophage exosomes as natural nanocarriers promote bone regeneration by targeting GATA2

**DOI:** 10.1093/rb/rbad075

**Published:** 2023-08-31

**Authors:** Xin Luo, Chunxiu Meng, Yujue Zhang, Qicui Du, Caiyao Hou, Huifen Qiang, Kun Liu, Zhaoyong Lv, Jun Li, Fengzhen Liu

**Affiliations:** Biomaterials Laboratory, Liaocheng People’s Hospital, Liaocheng Hospital affiliated to Shandong First Medical University, Liaocheng 252000, China; Biomaterials Laboratory, Liaocheng People’s Hospital, Liaocheng Hospital affiliated to Shandong First Medical University, Liaocheng 252000, China; Biomaterials Laboratory, Liaocheng People’s Hospital, Liaocheng Hospital affiliated to Shandong First Medical University, Liaocheng 252000, China; Biomaterials Laboratory, Liaocheng People’s Hospital, Liaocheng Hospital affiliated to Shandong First Medical University, Liaocheng 252000, China; Department of Materials Science and Engineering, Liaocheng University, Liaocheng 252000, China; Department of Materials Science and Engineering, Liaocheng University, Liaocheng 252000, China; Biomaterials Laboratory, Liaocheng People’s Hospital, Liaocheng Hospital affiliated to Shandong First Medical University, Liaocheng 252000, China; Biomaterials Laboratory, Liaocheng People’s Hospital, Liaocheng Hospital affiliated to Shandong First Medical University, Liaocheng 252000, China; Biomaterials Laboratory, Liaocheng People’s Hospital, Liaocheng Hospital affiliated to Shandong First Medical University, Liaocheng 252000, China; Biomaterials Laboratory, Liaocheng People’s Hospital, Liaocheng Hospital affiliated to Shandong First Medical University, Liaocheng 252000, China

**Keywords:** exosomes, miR-21a-5p, GATA2, macrophages, bone regeneration

## Abstract

Bone immune responses based on macrophages are critical in the osteogenesis of bone abnormalities. In general, M2 macrophage facilitate the promotion of osteogenesis, as well, M1 macrophage play an important role in early bone healing, as confirmed by previous studies. However, it is not clear how M1 macrophage are involved in the bone immune response. MiR-21a-5p is a highly expressed microRNA in M1 macrophage in contrast to M2. Therefore, the current work sought to ascertain the influence of M1 macrophage on bone healing via exosomal miR-21a-5p and the probable mechanism. We discovered that injecting M1 macrophage exosomes overexpressing miR-21a-5p into bone defect locations enhanced bone regeneration *in vivo*. Furthermore, by directly targeting GATA2, miR-21a-5p accelerated MC3T3-E1 osteogenic differentiation. Our findings showed that exosomal miR-21a-5p from M1 macrophage may be transported to osteoblasts and target GATA2 to enhance bone defect healing.

## Graphical abstract

**Graphical Abstract:**
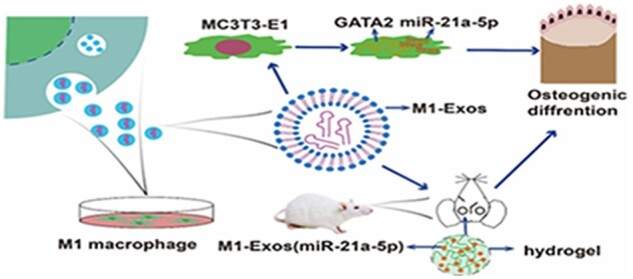

## Introduction

Skull and maxillofacial bone defects seriously affect the physiological function of patients due to tumors, trauma and various diseases, and how to repair bone defects is a clinical problem that needs to be solved [1, 2]. Currently, autologous bone graft is the most commonly used method in clinical practice; however, autologous bone graft has great limitations, such as large defect areas, insufficient donor area conditions and morphological mismatch [3]. In addition, allogeneic bone graft often causes immune rejection and infection, which leads to repair failure [4]. Therefore, new solutions are needed to promote bone healing and regeneration effectively. With the advancement of bone immunology in late years, increasing studies have shown the importance of the immune system for osteogenesis and repair, especially macrophages [5, 6]. Macrophages are plastic effector cells that are widely grouped into two phenotypes. Classically activated macrophages (M1), associated with cytokines secreted by T-helper type 1 (Th1) cells. Alternatively activated macrophages (M2), induced by T-helper type 2 (Th2) cells [7–9]. The balance between M1 and M2 is crucial during bone healing. In the beginning of ossification, M1 macrophage has a greater impact than M2 macrophage, while in the late stage, M2 macrophage promotes bone repair [10–12]. Qiao *et al*. [13] also demonstrated that the sequential activation pattern of macrophages phenotype during bone healing was important for biomaterial-induced bone regeneration. M1 macrophage caused osteoblastic recruiting to the damage area and were closely correlated with them on the new forming bone cover. Romero-López *et al*. [14] developed a 3D system for direct co-culture of mesenchymal stem cells (MSCs) and macrophage with different phenotypes and found that M1 macrophage enhanced bone formation in MSCs. Qu *et al*. [15] found that M1 macrophage derivative IL-6 accelerated the osteogenesis of ligamentum flavum cells. However, the factors controlling the useful effects of M1 macrophage in the setting of osteoblastic differentiation remain obscure.

Exosomes are natural nanocarriers (30–200 nm) with low immunogenicity, high biocompatibility and stable self-structure. In addition, as a form of extracellular vesicles, exosomes have various biological functions similar to those of their cells of origin and play an important role in cellular life activities, serving as a bridge between cells [16–18]. There is indication that macrophages play an important role in communication with neighboring cells through paracrine exosomes [19]. Wang *et al*. [20] showed that bone marrow mesenchymal stem cells (BMSCs) endocytosed macrophage-derived exosomes and that suppression of exosomes production dramatically reduced BMSC osteogenic recruitment mediated by macrophages. It has been demonstrated that engineered macrophage exosomes enriched with miR-3470b inhibited wear particle-induced osteolysis by suppressing TAB3/NF-κB *in vivo* [19]. Deng *et al*. [21] showed that the M2 macrophage-derived exosomal miR-590-3p reduced inflammatory signals and promoted epithelial regeneration by targeting LATS1 and subsequently activating YAP/β-catenin-regulated transcription. Exosomes contain significant amounts of non-coding RNAs, and microRNAs (miRNAs) are an important component [22]. Naked miRNAs are readily degraded by RNA enzymes in the blood or extracellular matrix during delivery to target tissues. The bilayer membrane structure of exosomes can ensure the stable presence of miRNA and further expand the specificity ability of the parent cell [23]. It was investigated that macrophage polarization leads to altered miRNAs in exosomes and can promote immune regulation during bone rejuvenation [24]. A separate report disclosed that the M2 macrophage-derived exosome miR-5106 targeted SIK2 and SIK3 genes to induce osteoblast differentiation [25]. Our previous study showed that M1 macrophage-derived extracellular vesicles overexpressed miR-21a-5p and promoted osteoblast differentiation of BMSCs [26]. However, the mechanisms controlling the beneficial effects of M1 macrophage exosomes in the context of osteoblast differentiation are still unknown. It has been demonstrated that exosomes can translocate enriched miRNAs to target cells and perform their functions by binding to the 3′ untranslated regions (UTR) of intracellular mRNAs leading to translational repression or target degradation [27]. Herein, we present evidence that M1 macrophage-derived exosome miR-21a-5p is targeted to GATA2 to promote bone healing. Thus, the data can suggest that exosomal miR-21a-5p will be a novel treatment tactic for bone defect repair.

## Materials and methods

### Cell culture

RAW 264.7 and MC3T3-E1 cells were obtained from american type culture collection (ATCC). RAW 264.7 and MC3T3-E1 cell complete medium were prepared by mixing dulbecco's modification of eagle's medium (DMEM) (Gibco) and α-MEM (Gibco) with 10% heat-inactivated fetal bovine serum (Gibco), respectively, along with 1% penicillin/streptomycin (P/s). The osteoinduction medium was configured by adding 100 μg/ml ascorbic acid, 10 mM β-glycerophosphate and 10 mM dexamethasone to α-MEM complete medium. The reagents were obtained from sigma. RAW 264.7 cells (M0) were treated with 100 ng/ml lipopolysaccharide and 20 ng/ml interferon-γ for 24 h as previously described and induced to M1 type, while treatment with 20 ng/ml interleukin-4 for 24 h induced to M2 type [26]. Cells were incubated in a cell culture incubator containing 5% CO_2_ at 37°C.

### Cell co-culture system

Exosomes secreted by macrophages of different phenotypes were dissolved into an osteogenic induction medium (1 μg/ml) and named as M0-Exos, M1-Exos and M2-Exos, respectively. The control group was the osteogenic induction medium. A 24-well plate was inoculated with 2 × 10^5^ MC3T3-E1 cells per well, and the complete medium was replaced with an osteogenic induction medium prepared with different exosomes after 24 h. The liquid was altered every 2 days. On 7 days, qRT-PCR was performed to measure the expression of osteogenic genes. The miR-21a-5p was knocked down and overexpressed in RAW 264.7 cells using a lentiviral vector, after which it was induced to become M1 type and exosomes were extracted, named sh-miR-21a-5p and OE-miR-21a-5p. The extracellular vesicles were supplemented to the osteogenesis induction medium of MC3T3-E1 cells, respectively. Osteoblastic differentiation of MC3T3-E1 was induced according to the same method described above, and osteogenic genes were identified.

### QRT-PCR

Total RNA was obtained from MC3T3-E1 using Trizol (Takara). The M-MuLV First Strand cDNA Synthesis Kit and miRNA first Strand cDNA Synthesis (Sangon Biotech) was applied to produce cDNA. The 2 × SG Fast qPCR Master Mix was used to detect relative mRNA or miRNA levels on a system following the manufacturer. Quantified by the 2-ΔΔCt method. Glyceraldehyde-3-phosphate dehydrogenase (GAPDH) and U6 were applied as controls. All sequences of primers were presented in Table 1.

**Table 1.** Primer sequences

Gene	Primer	Sequences (5′–3′)
*COL-1*	Forward	GTGGCGGTTATGACTTCAGC
	Reverse	TCACGAACCACGTTAGCATC
*Runx2*	Forward	AAATGCCTCCGCTGTTATGAA
	Reverse	GCTCCGGCCCACAAATCT
*OCN*	Forward	CCGGGAGCAGTGTGAGCTTA
	Reverse	AGGCGGTCTTCAAGCCATACT
*BMP-2*	Forward	TGACTGGATCGTGGCACCTC
	Reverse	CAGAGTCTGCACTATGGCATGGTTA
*ALP*	Forward	AGGGTGGGTAGTCATTTGCATAG
	Reverse	GAGGCATACGCCATCACATG
*OPN*	Forward	ATCTCACCATTCGGATGAGTCT
	Reverse	TGTAGGGACGATTGGAGTGAAA
*GATA2*	Forward	GCTCTAGAATGGAGGTGGCGCCTGAGCAGCC
	Reverse	CCGCTCGAGCTAGCCCATGGCAGTCACCATGC
*miR-21a-5p*	Forward	CGCTAG CTTATCAGAC TGA
	Reverse	CTCAACTGGTGTCGTGGA
*U6*	Forward	CTCGCTTCGGCAGCACA
	Reverse	AACGCTTCACGAATTTGCGT
*GAPDH*	Forward	TGACCACAGTCCATGCCATC
	Reverse	GACGGACACATTGGGGGTAG

### Exosomes release curve

Macrophage-derived exosomes were extracted as previously described [12]. The HyStem-HP Hydrogel Kit (Advanced BioMatrix) was opened in an ultra-clean table and the mixture of hydrogel and exosomes was prepared according to the instructions. The mixture was transferred to 96 plates with 100 μl per well and incubated for 1 h to prepare the materials. Phosphate Buffer Solution (PBS) was then placed in the 96-well plate to submerge hydrogel, which was left for 14 days and PBS was collected from the submerged materials at the same time each day. Protein content in PBS was assayed according to the BCA Protein Assay Kit (Beyotime) instructions, thus plotting the slow-release curve of exosomes.

### Scanning electron microscope

Hydrogels loaded with M1 exosomes and M1 exosomes overexpressing miR-21a-5p were stored at −80°C for 12 h and then freeze-dried. The freeze-dried samples were sputtered with gold for 1.5 min under vacuum to ensure the conductivity of the samples and then the exosomes were observed using scanning electron microscope (SEM).

### Rat cranial defect model and treatment

**Figure 1.**
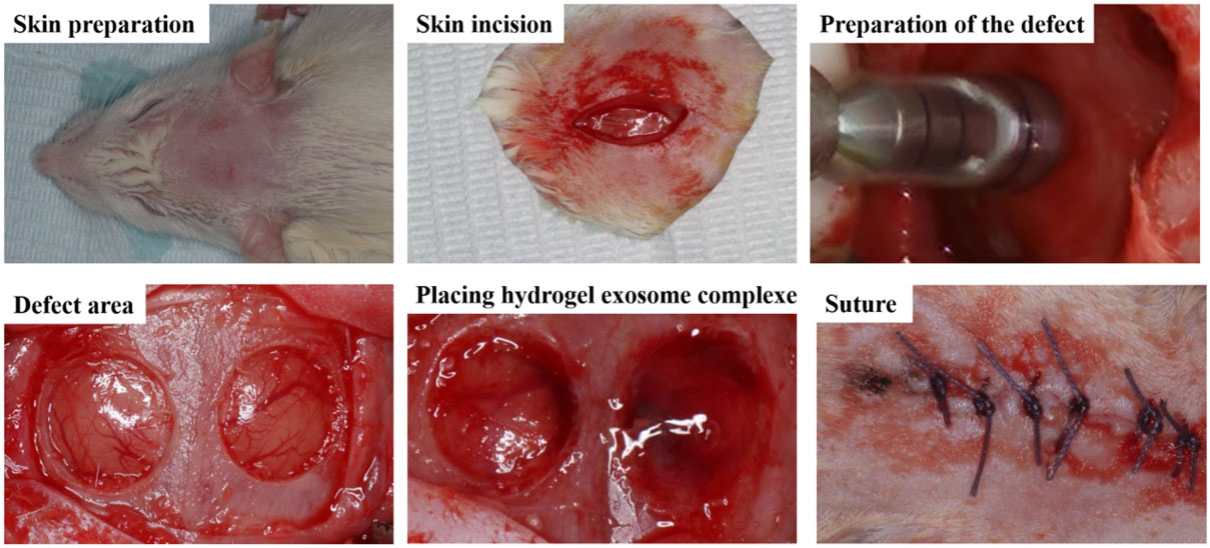
Preparation of rat cranial defect model.

Augmentation of 8-week-old male Sprague–Dawley rats by intraperitoneal injection with 1% sodium pentobarbital (30 mg/kg) followed by the creation of bilateral transcortical defects using a 5.0-mm trephine dental drill under saline solution irrigation. Twenty-seven rats were randomly assigned, and all defects on the left side were control group without any treatment. The defects on the right side were filled with hydrogel (hydrogel group), hydrogel loaded with M1 macrophage exosomes (M1-Exos group) and hydrogel loaded with M1 macrophage exosomes overexpressing miR-21a-5p (M1-Exos-miR-21a-5p group). The wound was then closed in layers followed by topical application of erythromycin ointment and intramuscular injection of penicillin 400 000 units. The animals were free to get their meals and fluid after surgery, while the status of the rats was closely observed. Cranial bone was taken at 2, 4 and 8 weeks after surgery and secured in 4% formalin for 1 d before histological and imaging analysis. The ethics committee of Liaocheng People’s Hospital approved and agreed on the protocol of the trial (2021019). The experimental procedure was shown in Figure 1.

### Cone beam CT analyses

The skulls of rats were collected for cone beam CT (CBCT) (ORTHOPHOS XG 3D) analysis. Image acquisition was performed at 85 kV and 4 mA with 360° rotation between frames and 14.4 s exposure time. CBCT reconstruction software was used to examine bone defect healing quality and assess bone volume fraction (BV/TV).

### HE and Masson staining

Rat cranial bone was de-calcified in 10% ethylenediamine tetraacetic acid (EDTA) solution for 6 weeks and then encapsulated in paraffin and cut into 5-μm sections. The slices were dewaxed in xylene and entered into distilled water through a gradient of alcohol concentration from high to low. The cuts were stained according to the instructions of the Hematoxylin and Eosin Staining Kit (Beyotime) and Masson’s Trichrome Stain Kit (Solarbio). The stained sections were dehydrated at a gradient of alcohol concentration from low to high, then the slices were clarified by xylene and coated with neutral gum.

### Immunohistochemical staining

The cuts were dewaxed into water. Incubated for 10 min in volume fraction 3% hydrogen peroxide deionized water (avoid light) to eliminate endogenous peroxidase activity. The antigen repair was treated with pepsin and incubated for 40 min at 37°C. The cuts were closed with goat serum working solution and placed for 30 min. Primary antibody anti-osteocalcin, overnight at 4°C and incubated for 30 min at 37°C with secondary antibody. 3,3’-Diaminobenzidine (DAB) working solution was used for color development, and hematoxylin was applied for re-staining the nucleus. Osteocalcin showed brownish-yellow particles in the cytoplasm as a positive cell marker. Immunohistochemical staining images were analyzed with Image pro plus 6.0 software.

### Lentiviral transduction

The miR-21a-5p knockdown and overexpression lentivirus and GATA2 overexpression lentivirus were obtained from Shanghai Genechem Co., LTD. The miR-21a-5p was knocked down and overexpressed in RAW 264.7 cells according to the method described previously [26]. MC3T3-E1 cells were infected with miR-21a-5p overexpression lentivirus and GATA2 overexpression lentivirus, respectively. Afterward, positive cells were screened with puromycin, and positive cells (green fluorescence) were observed under a fluorescence microscope after 1 week. MC3T3-E1 cells overexpressing miR-21a-5p were re-infected with GATA2 overexpressing lentivirus in the same way.

### ALP and ARS staining

ALP staining and activity were achieved with the P-toluidine salt (BCIP)/nitroblue tetrazole (NBT) Alkaline Phosphatase Color Development Kit (Beyotime) and Alkaline Phosphatase Assay Kit (Beyotime) following the fabricant’s directions.

For ARS dying, MC3T3-E1 were immobilized with 4% paraformaldehyde for 40 min followed by ddH_2_O washed three times, and stained with Alizarin Red S Solution (Solarbio) for 40 min. The mineralized nodules were dissolved with 2% cetylpyridinium chloride solution (Sigma), and then the dissolved solution was moved to a 96-well plate and the absorbance at 562 nm was calculated for quantitative analysis.

### Western blot analysis

Lysis of cells with RIPA (Beyotime) containing Phenylmethanesulfonyl fluoride (PMSF) (Beyotime) to extract total protein. The protein concentration was measured by a BCA kit (Beyotime). Proteins were isolated by sodium dodecyl dulfate polyAcrylamide gel electrophoresis (SDS-PAGE) and moved to polyvinylidene fluoride (PVDF) membrane (Bio-Rad), which was then closed with 5% skim milk for 70 min. Incubate the film with GATA2 (Abcam) and GAPDH (Abcam) primary antibody overnight at 4°C. The films were further reacted with the secondary antibody solution (Beyotime) for 70 min. Signals were captured and quantitated by the Bio Rad chemiluminescence system.

### Enzyme analysis

Mixed 2 µl plasmid, 1 µl Buffer Smart, 1 µl SacI restriction endonuclease, 1 µl MluI restriction endonuclease and 5 µl deionized water in a tube. Afterward, the tubes were placed in a PCR instrument and the reaction conditions were set at 37°C for 60 min. After the reaction, the tubes were subjected to agarose gel electrophoresis to observe whether double bands were formed after enzymatic digestion.

### Luciferase reporter assay

MC3T3-E1 cells and MC3T3-E1 cells overexpressing miR-21a-5p were transfected with pMIR-GATA2-wt and pMIR-GATA2-must plasmids (Sangon Biotech) using X-tremegene HP (Roche). The lysates were collected within 48 h and luciferase activity was determined in the Dual-Luciferase^®^ Reporter Assay System (Promega) instructions. Standardized sea kidney fluorophore luciferase activity.

### Statistical analysis

The data were analyzed using GraphPad Prism 9.0. All data were repeated three times and expressed as mean ± SD. Comparisons between the two groups were made using a two-tailed Student’s *t*-test and *P*-values <0.05 were considered statistically significant.

## Results

### M1 macrophage exosome miR-21a-5p promoted MC3T3-E1 osteogenic differentiation

**Figure 2.**
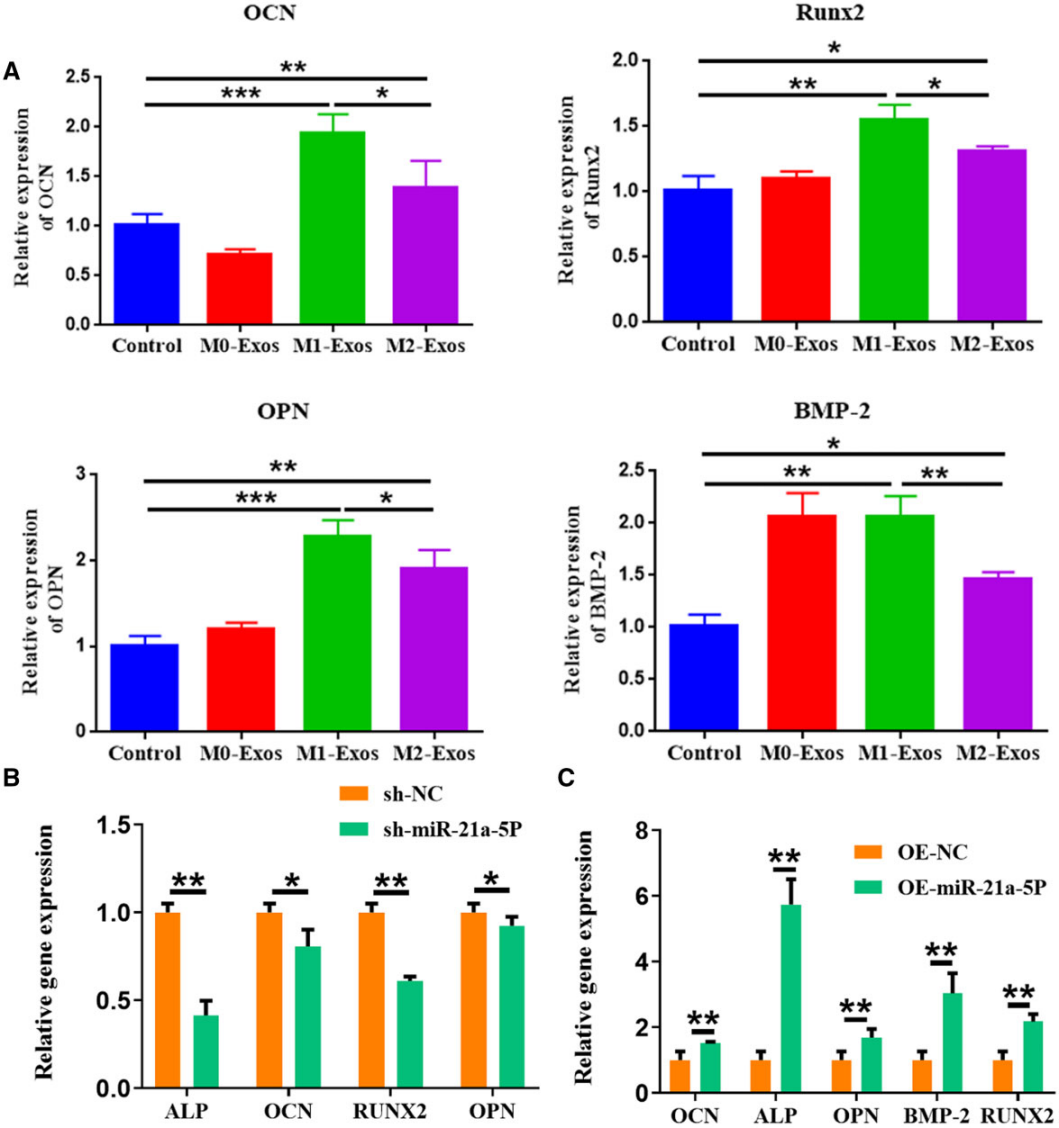
M1 macrophage exosome miR-21a-5p promoted MC3T3-E1 osteogenic differentiation. (**A**) M1 macrophage exosome promoted osteogenesis-related gene expression in MC3T3-E1, (**B**) Knockdown of M1 macrophage exosome miR-21a-5p and inhibition of osteogenesis-related gene expression in MC3T3-E1 cells, (**C**) Overexpression of M1 macrophage exosome miR-21a-5p promoted the expression of osteogenic-related molecules in MC3T3-E1 cells. *P < 0.05, **P < 0.01, ***P < 0.001.

Our previous study showed that miR-21a-5p was abundant in exosomes derived from M1 macrophage and promoted osteogenic differentiation of osteoblasts [22]. To investigate the mechanism by which M1 macrophage-derived exosomes promoted bone repair, we investigated the influence of M1 macrophage exosome and M1 macrophage exosome miR-21a-5p on osteoblast differentiation of MC3T3-E1. M0, M1 and M2 macrophage exosomes were co-cultured with MC3T3-E1, respectively, and the expression of osteogenic genes was examined by qRT-PCR. The expression of OCN, OPN, Runx2 and BMP2 mRNA was found to be the most significant in the M1-Exos group (Figure 2A). We further investigated the influence of M1 macrophage exosome miR-21a-5p on MC3T3-E1 osteoblast differentiation. We knocked down and overexpressed miR-21a-5p in M1 macrophage, extracted exosomes and co-cultured them with MC3T3-E1, and detected osteogenic gene expression by qRT-PCR. The findings showed that knockdown of miR-21a-5p inhibited osteogenic gene expression and overexpression of miR-21a-5p promoted osteogenic gene expression (Figure 2B and C).

### M1 macrophage exosome miR-21a-5p accelerated rat cranial defect healing

**Figure 3.**
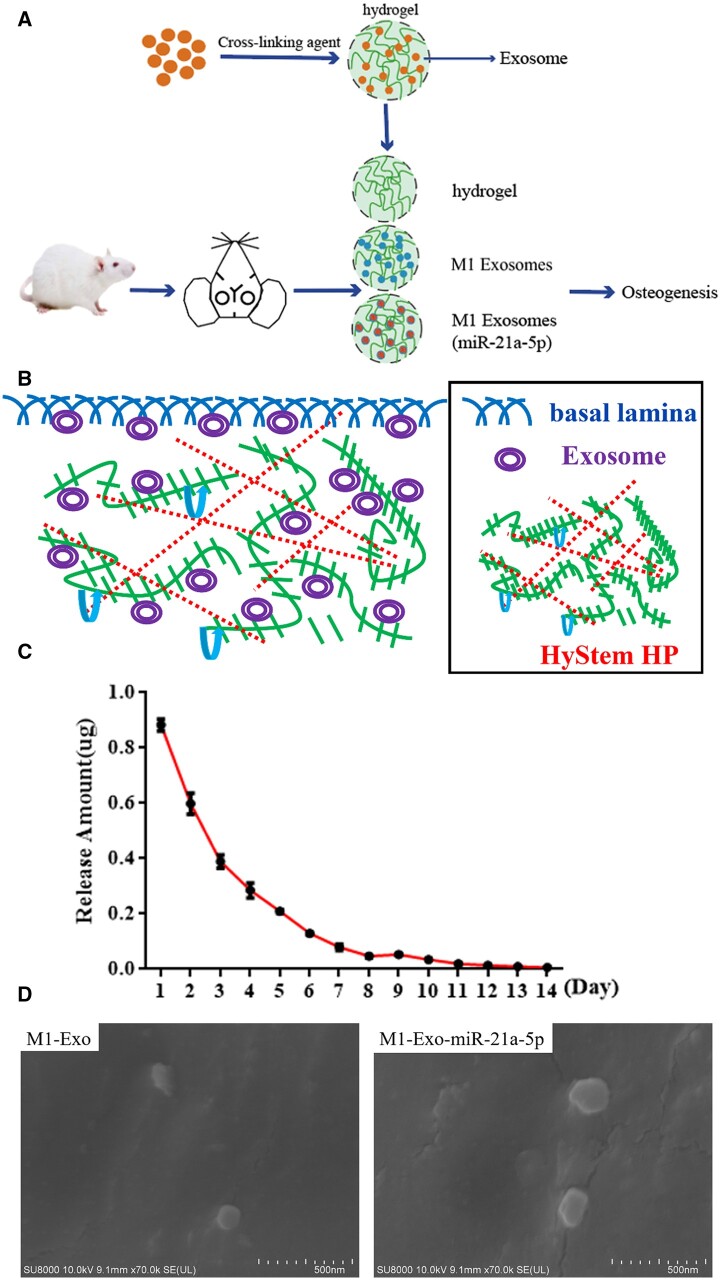
MiR-21a-5p-modified M1 macrophage exosomes complexed with hydrogel for the treatment of skull defects in rats. (**A**) Model diagram of rat cranial defect experiment, (**B**) Crosslinking pattern diagram of hydrogel and exosomes, (**C**) Exosome release curve, (**D**) Observations of hydrogel-loaded M1 macrophage exosomes and M1 macrophage exosomes overexpressing miR-21a-5p by SEM (scale: 500 nm).

The rapid clearance of exosomes in the body and their lack of tissue targeting limit their therapeutic potential. HyStem™ hydrogels (Merck) closely mimic the natural environment of the extracellular matrix. We used hydrogels to encapsulate exosomes and improved their retention in the body, thereby enhancing their therapeutic efficacy. As shown in Figure 3A, a rat cranial defect model was constructed, in which hydrogel-loaded M1 macrophage exosomes and M1 macrophage exosomes overexpressing miR-21a-5p were placed in the defect. Figure 3B showed the cross-linking pattern of hydrogel and exosomes. To detect the rate of exosomes release in the hydrogel, the exosome-loaded hydrogel was placed in physiological saline. The results showed that the exosomes were stable and slowly released in the hydrogel for about 14 days (Figure 3C). SEM images showed that exosomes were distributed on the hydrogel skeleton (Figure 3D).

**Figure 4.**
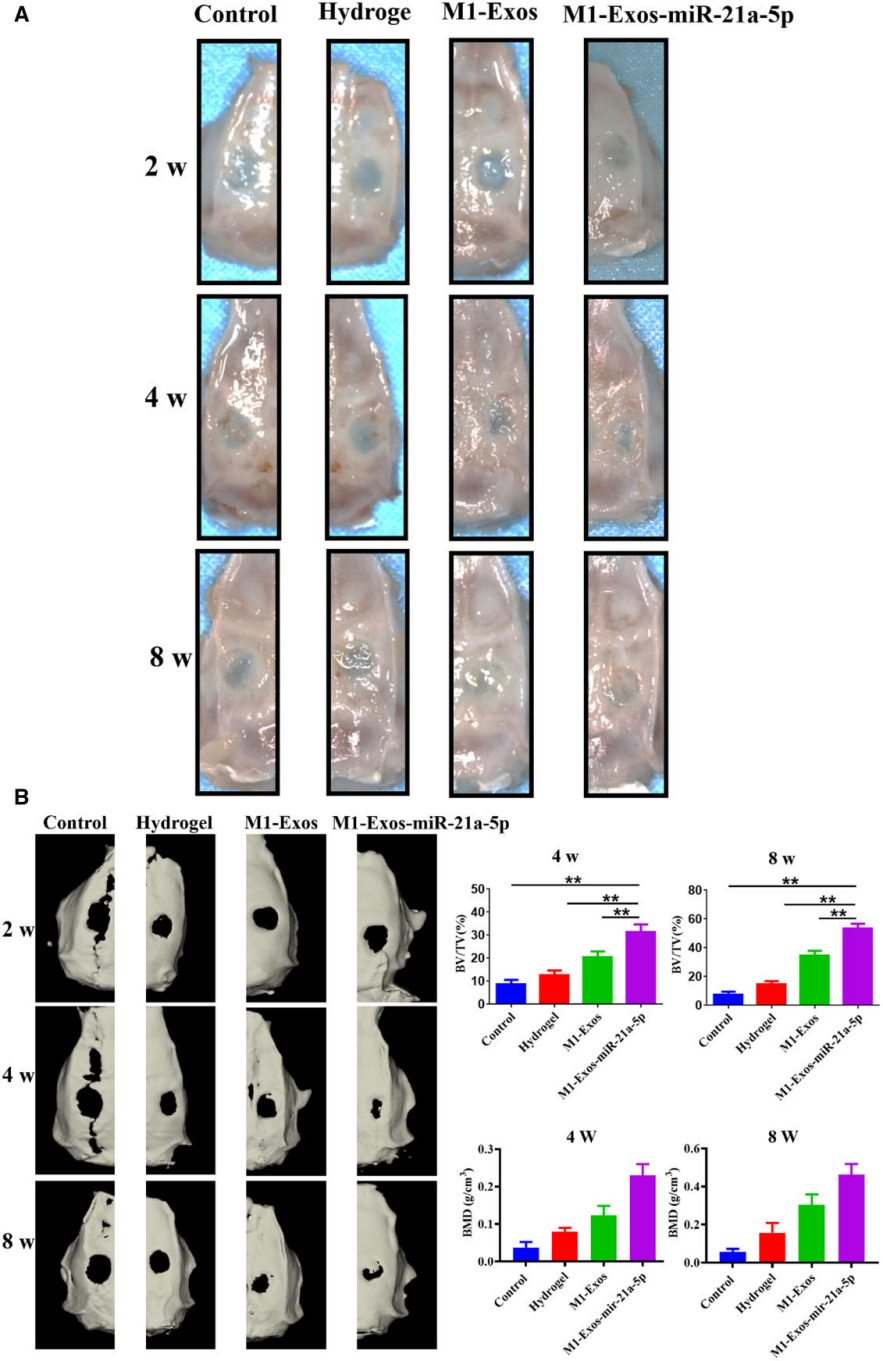
Gross observation and CBCT scan of the skull defect in rats. (**A**) Cranial gross observation of rats at 2, 4 and 8 weeks after operation, (**B**) CBCT 3D reconstruction and volume quantification of novel bone at 2, 4 and 8 weeks after operation. **P < 0.01.

Gross observation and CBCT scans were performed at 2, 4 and 8 weeks post-operatively to monitor the healing process of the bone defects. The results were displayed in Figure 4, with no significant changes in the area of the skull defects in the four groups after 2 weeks. At 4 and 8 weeks, the high-density shadow area was dramatically increased in the M1-Exos-miR-21a-5p group. In addition, the M1-Exos-miR-21a-5p group had significantly higher BV/TV and bonemineraldensity (BMD). Furthermore, M1 exosomes overexpressing miR-21a-5p composited with hydrogel showed excellent osteogenic potentiality [12].

**Figure 5.**
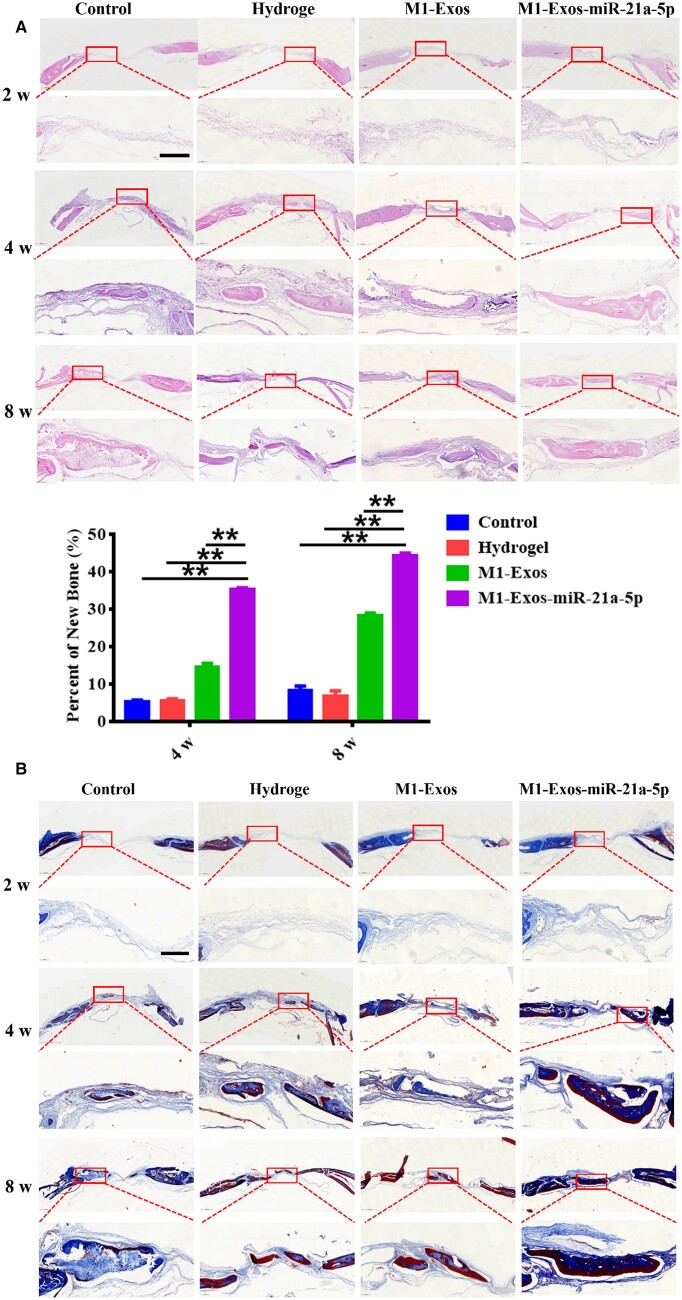
HE and Masson staining of skull defects in rats. (**A**) Qualitative and quantitative analysis of HE staining of the defect at 2, 4 and 8 weeks after operation (scale: 100 μm), (B) Masson staining of the defect at 2, 4 and 8 weeks after operation (scale: 100 μm). **P < 0.01.

HE staining showed that after 2 weeks, both ends of the defect area in all four groups were connected by a large amount of fibrous connective tissue, and no new bone was seen. After 4 and 8 weeks, regenerated bone was observed in the defect area of M1-Exos and M1-Exos-miR-21a-5p groups, and connective tissue was filled between new bone and old bone. The M1-Exos-miR-21a-5p group showed the most significant increase in bone area with dense new bone in the center of the deficiency area. Quantitative analysis showed a higher percentage of new bone creation in the M1-Exos-miR-21a-5p group (Figure 5A). Masson staining showed that after 2 weeks, no new bone was seen in the deficiency area in any of the four groups. After 4 and 8 weeks, the defects in M1-Exos and M1-Exos-miR-21a-5p groups were surrounded by blue new bone tissue and red mature bone tissue, and new bone formation was more obvious in M1-Exos-miR-21a-5p group (Figure 5B).

**Figure 6.**
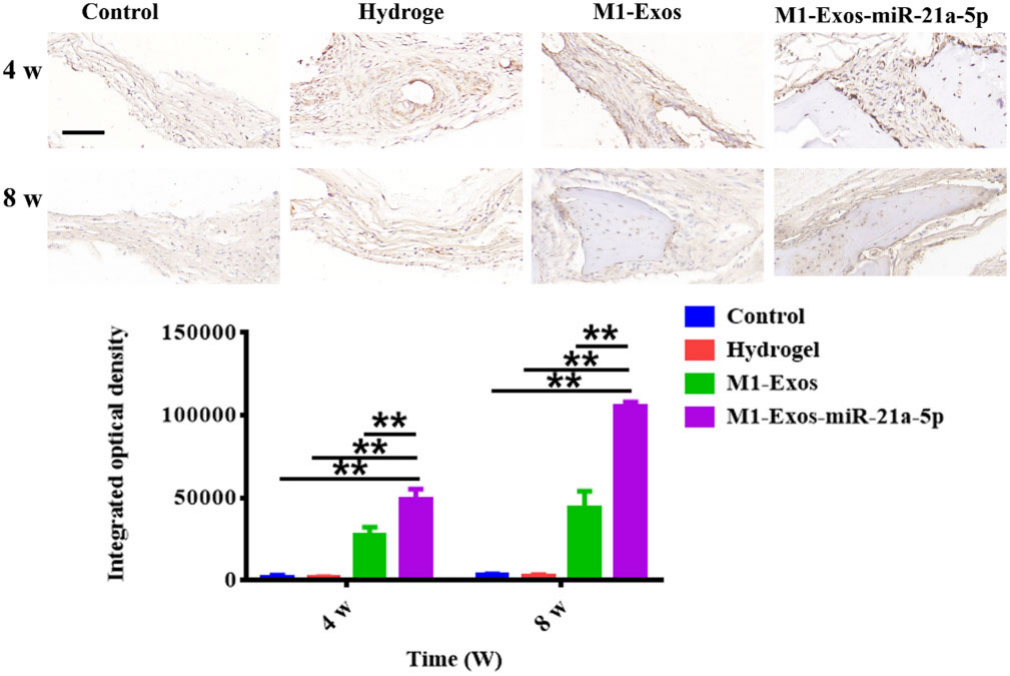
Comparison of osteocalcin immunohistochemical staining and IOD in the cranial defects of rats at 4 and 8 weeks after surgery (scale: 50 μm). **P < 0.01.

Immunohistochemical staining showed a major increase in OCN expression in the M1-Exos-miR-21a-5p group at 4 and 8 weeks (Figure 6). It was suggested that miR-21a-5p derivative from M1 exosomes promoted bone defect healing.

### MiR-21a-5p induced MC3T3-E1 osteoblast differentiation

**Figure 7.**
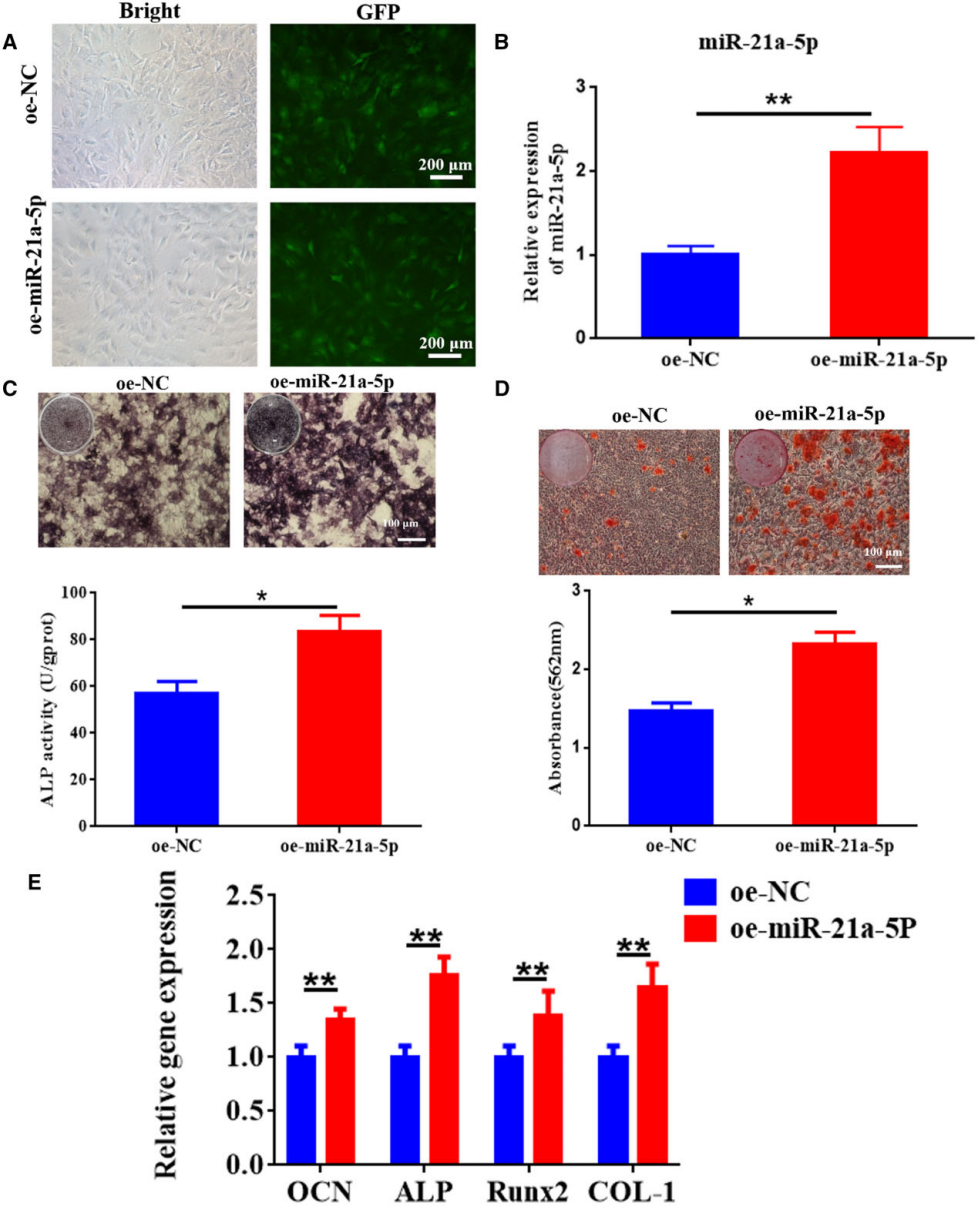
MiR-21a-5p induced MC3T3-E1 osteoblast differentiation *in vitro*. (**A**) Microscopic observation of fluorescent expression of lentivirus after transfection (scale: 200 μm). (**B**) Gene expression of miR-21a-5p was detected by qRT-PCR. (**C**) ALP staining and activity in MC3T3-E1 (scale: 100 μm). (**D**) Alizarin red S staining and quantification in MC3T3-E1 (scale: 100 μm). (**E**) Osteogenesis-related genes were determined by qRT-PCR. *P < 0.05, **P < 0.01.

Next, we explored the ability of miR-21a-5p to directly affect MC3T3-E1 by treating cells with miR-21a-5p overexpressing lentivirus and showed that infection with lentivirus significantly increased the expression of miR-21a-5p in MC3T3-E1 (Figure 7A and B). Effect of miR-21a-5p on osteogenic differentiation of MC3T3-E1 as detected by staining with ALP, staining with alizarin red, and assaying osteogenic gene expression. The outcomes indicated that miR-21a-5p induced more significant ALP activity and staining (Figure 7C). Alizarin red staining showed mineral sedimentation was increased in the oe-miR-21a-5p group (Figure 7D). Moreover, osteogenesis-related genes COL-1, ALP, OCN and Runx2 were significantly increased in the oe-miR-21a-5p group (Figure 7E). Together, these results validated the ability of miR-21a-5p to directly induce ossification of MC3T3-E1.

### GATA2 was miR-21a-5p target gene

According to the database (http://www.targetscan.org) [28], miR-21a-5p could directly bind the 3′UTR of GATA2 and GATA2 is a transcription factor of ALP. In addition, GATA2 negatively regulates osteogenic differentiation [29] (Figure 8A and B). Overexpression of miR-21a-5p in MC3T3-E1 and detection of GATA2 expression by qRT-PCR and western blot revealed that miR-21a-5p reduced GATA2 mRNA and protein expression (Figure 8C and D). Specific conjugation among miR-21a-5p and GATA2 was established by a dual luciferase reporter assay. The pMIR vector bound the 3′UTR of wild-type and mutant GATA2 and transfected MC3T3-E1 cells overexpressing miR-21a-5p. The outcomes indicated that mutating the 3′UTR region of GATA2, miR-21a-5p was no longer found to bind to inhibit fluorophore enzyme activity (Figure 8E and F).

**Figure 8.**
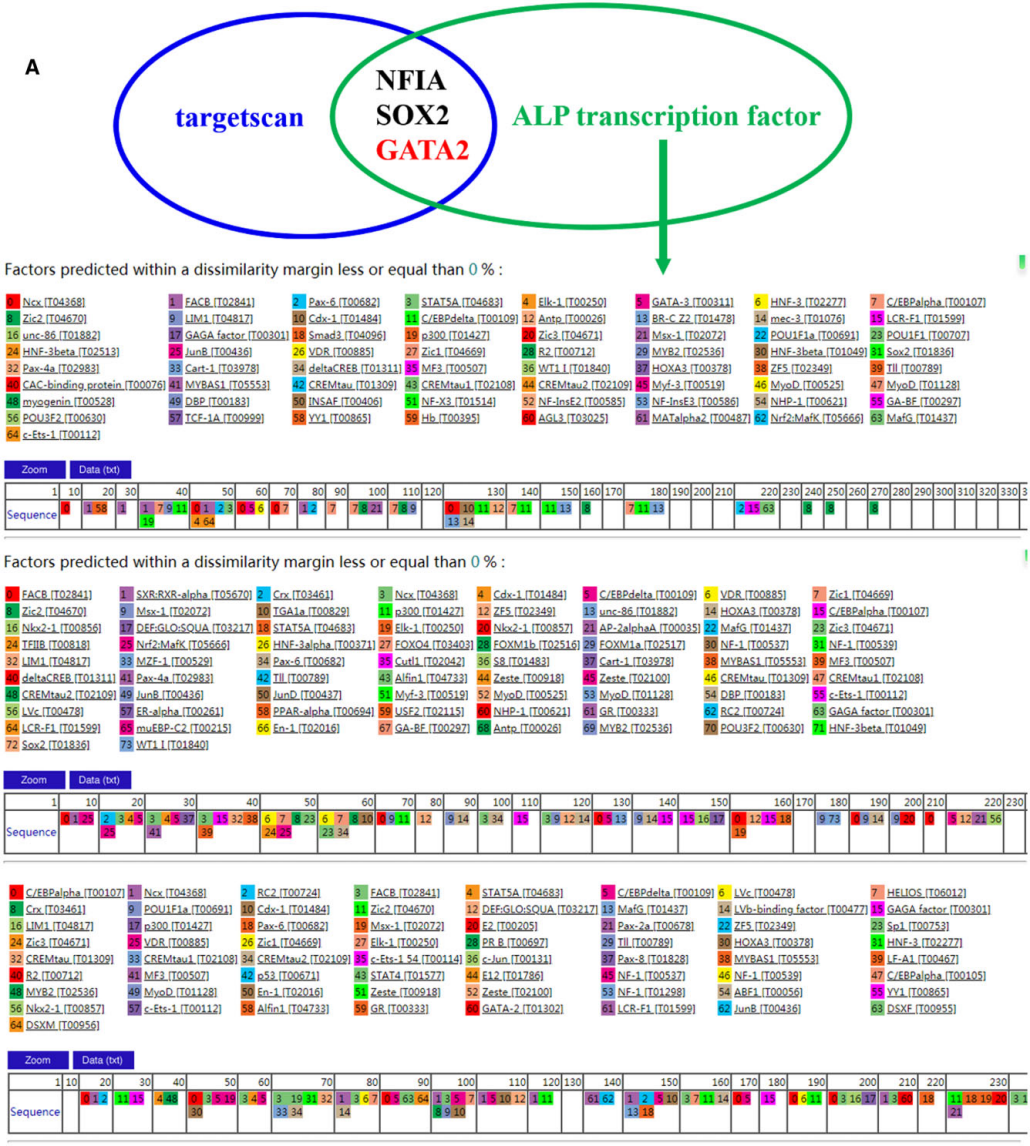
GATA2 was miR-21a-5p target gene. (**A**) Targetscan analysis and ALP upstream transcription factor intersection, (**B**) Luciferase reporter constructs containing either a WT GATA2 3′ UTR or the same region after site-directed mutagenesis, (**C**) Detection of GATA2 mRNA expression in MC3T3-E1 after overexpression of miR-21a-5p by qRT-PCR, (**D**) Western blot detection of GATA2 expression in MC3T3-E1 after overexpression of miR-21a-5P, (**E**) Enzymatic validation of pMIR-GATA2-wt and pMIR-GATA2-mut, (**F**) Target relation of miR-21a-5p and GATA2 ascertained by dual luciferase reporter assay. *P < 0.05, **P < 0.01.

**Figure 8.**
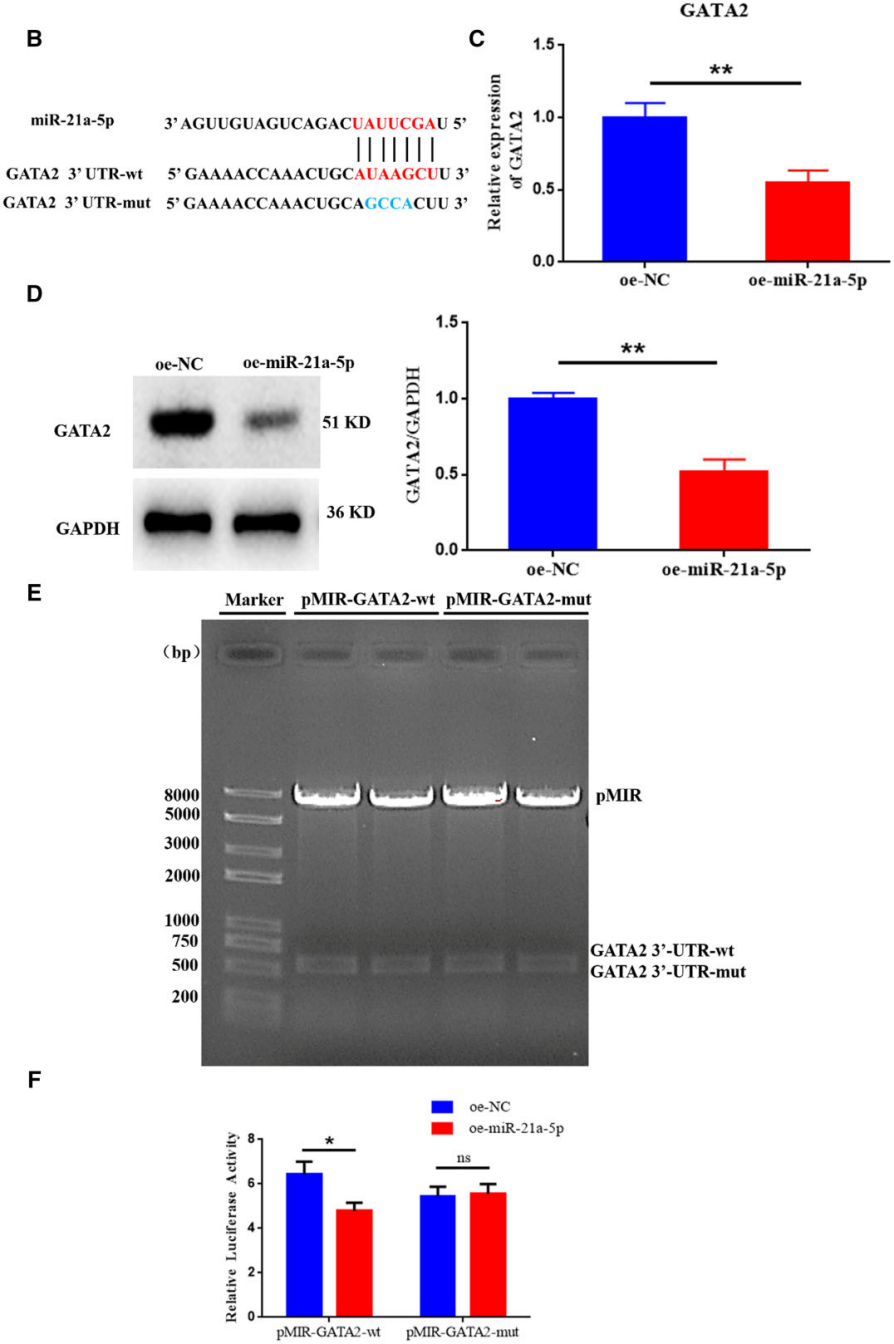
(Continued).

### MiR-21a-5p targeted GATA2 to induce osteoblast activity *in vitro*

To explore the dependence of osteogenic differentiation of MC3T3-E1 cells on GATA2, we overexpressed both miR-21a-5p and GATA2 to detect osteogenic differentiation of MC3T3-E1. First, GATA2 was overexpressed in MC3T3-E1 cells using lentivirus, and its expression was detected by qRT-PCR, which revealed a significantly high expression of GATA2 in the oe-GATA2 group (Figure 9A and B). Second, it was found that miR-21a-5p and GATA2 were significantly highly expressed in MC3T3-E1 after lentiviral transfection, and overexpression of miR-21a-5p drastically diminished the expression of GATA2 (Figure 9C and D). ALP staining and activity analysis revealed that ALP expression was reduced in MC3T3-E1 when GATA2 was overexpressed, and overexpression of miR-21a-5p partially rescued their negative effect on osteogenic differentiation (Figure 9E). When GATA2 was overexpressed in MC3T3-E1, calcium nodule and osteogenic-related gene (COL-1, ALP and OCN) expression were reduced in MC3T3-E1, and overexpression of miR-21a-5p partially rescued their negative effects on osteogenic differentiation in MC3T3-E1 (Figure 9F and G). It was a diagram of miR-21a-5p regulation of MC3T3-E1 osteogenic differentiation pattern in Figure 9H.

**Figure 9.**
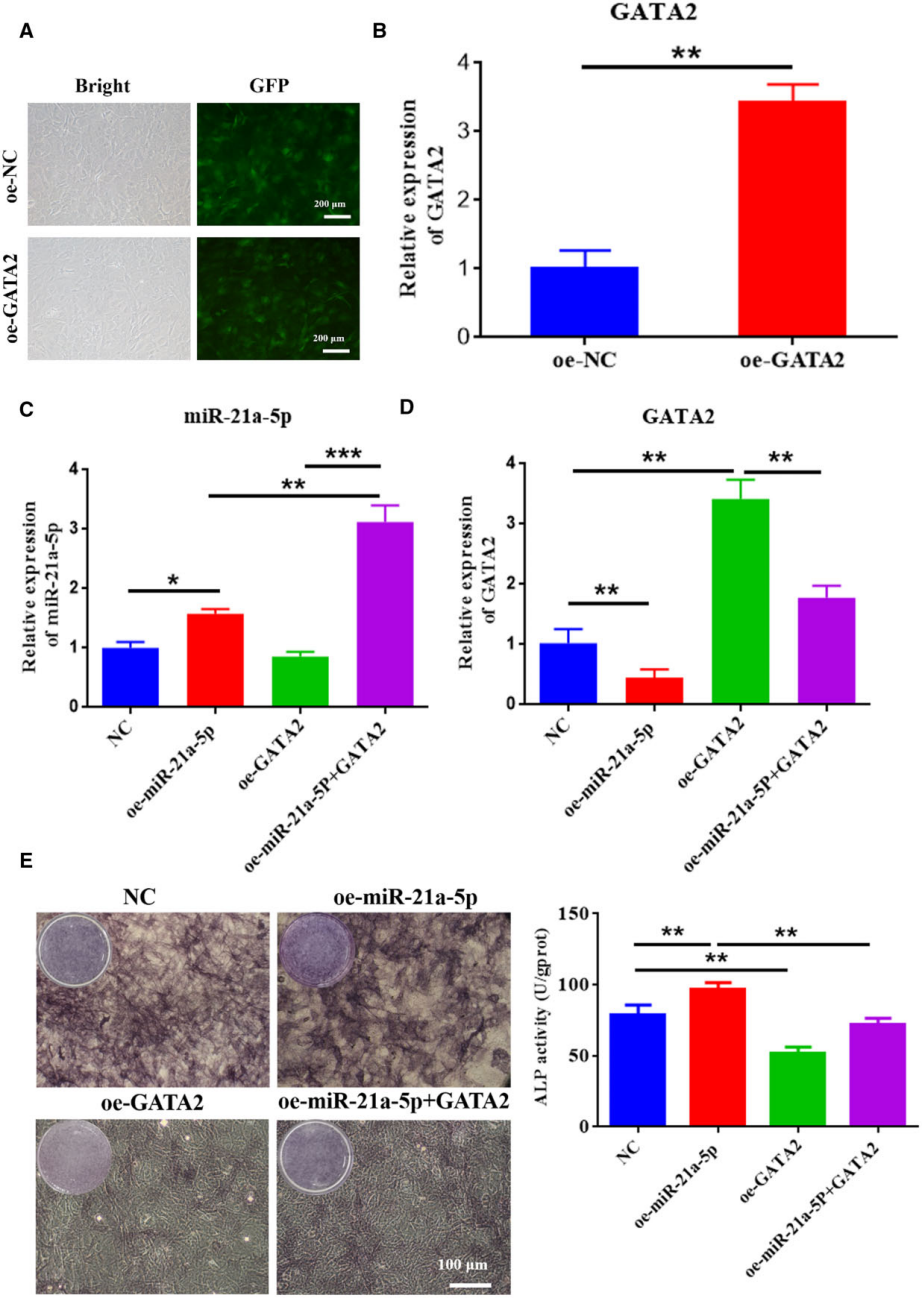
MiR-21a-5p targeted GATA2 to induce osteoblast activity *in vitro*. (**A**) Fluorescence microscopy of infection efficiency of overexpressed GATA2 lentivirus (scale: 200 μm), (**B**) Overexpression of GATA2 in MC3T3-E1 cells verified by qRT-PCR, (**C**) Revelation of miR-21a-5p expression in MC3T3-E1 cells by qRT-PCR, (**D**) Detection of GATA2 expression in MC3T3-E1 cells by qRT-PCR, (**E**) ALP staining and activity assay (scale: 100 μm), (**F**) ARS staining and quantitative assay (scale: 100 μm), (**G**) Detection of MC3T3-E1 osteogenesis-related gene expression by qRT-PCR, (**H**) The pattern of miR-21a-5p regulation of MC3T3-E1 osteogenic differentiation. *P < 0.05, **P < 0.01, ***P < 0.001.

**Figure 9.**
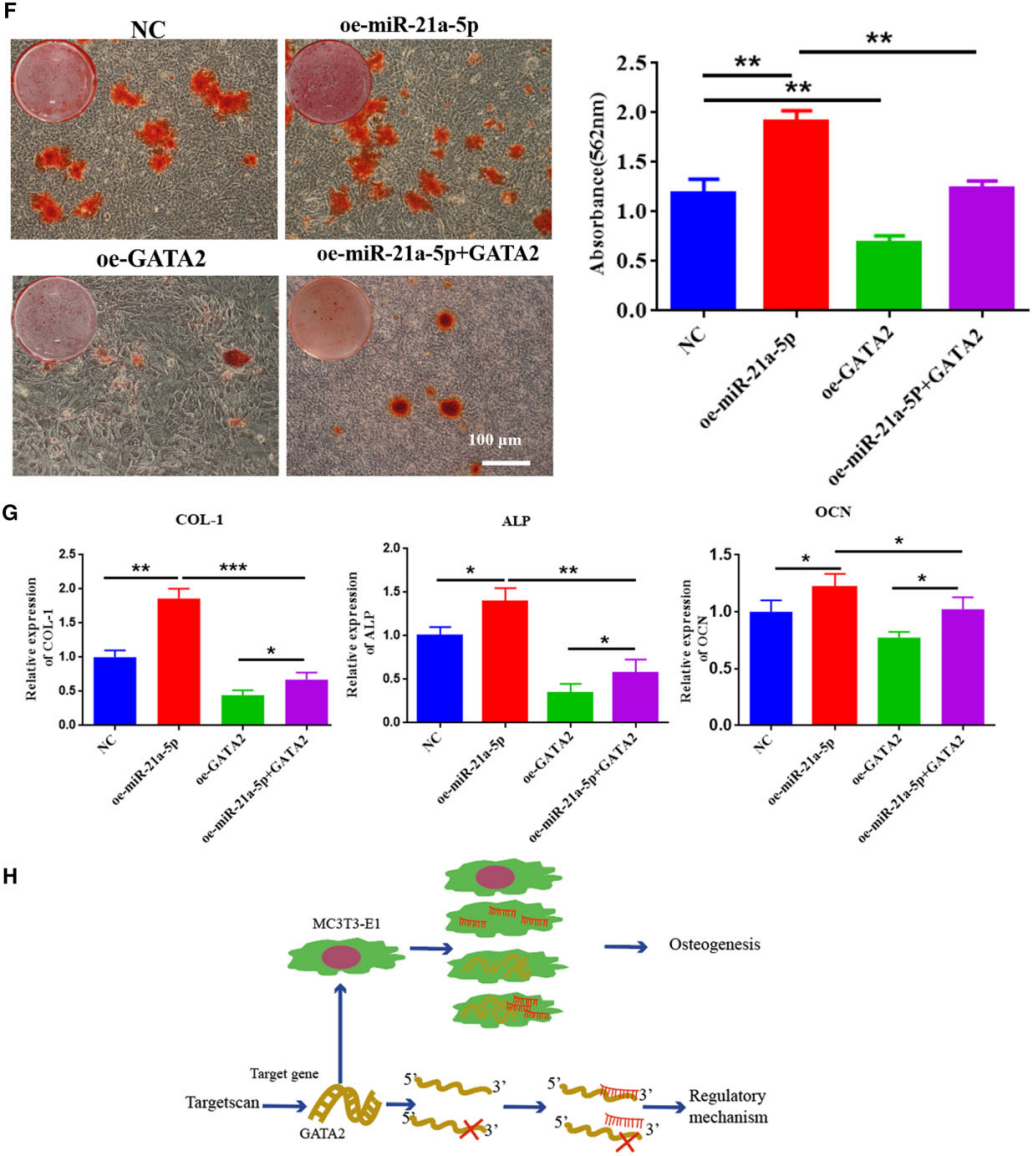
(Continued).

## Discussion

Biomaterials have great promise for bone restoration purposes as an alternative to bone implants. Previously the use of barrier membranes to exclude non-osteogenic tissue was the gold standard for guiding bone regeneration, as rapidly proliferating epithelium and connective tissue interfered with bone regeneration. However, research has focused on optimizing the osteogenic capacity of biomaterials at the expense of investigating the immune response that they trigger [30, 31]. In recent years, there has been a hot spot for osteoimmunology, which studies the fundamental communication between the immune and skeletal systems [32]. Immune cells are involved in the regulation of bone deposition and bone strength by influencing the activity of osteoblasts, osteoclasts and osteocytes [33]. Although multiple immune cells are involved in the immune response in bone tissue, macrophages perform the most vital function by secreting various cytokines. Macrophages can achieve dynamic regulatory effects on different bone repair stages through the interconversion of M1 and M2 types [14, 34]. Nathan *et al*. [35] suggested that M1 macrophage was essential for optimal matrix mineralization at 72–96 h. Li *et al*. demonstrated that mineralized collagen with 84 μm pore size promoted macrophage polarization toward M2 and mediated bone immunity for bone regeneration.

Presently, exosomes are emerging as the new form of ‘cell-free therapy’ that can serve an important therapeutic role in promoting wound healing and bone tissue regeneration [18]. Both immune cells and bone repair cells are capable of releasing exosomes, which are non-homogeneous vesicular structures at the nanoscale. Because exosomes can exchange lipids, proteins and nucleic acids between cells through cellular and molecular mechanisms, they are able to exchange intercellular information and bioactive components between the skeletal and immune systems [36, 37]. Liu *et al*. [38] used strontium-substituted calcium silicate to stimulate BMSCs-derived exosomes (Sr-CS-Exo). The results revealed that Sr-CS-Exo had superior pro-angiogenic ability and contributed to accelerated developmental angiogenesis in zebrafish, as well as neovascularization and bone regeneration in distal femoral defects in rats. Liu *et al*. [39] used mesoporous bioactive glass to slow release BMSC-derived exosomes at rat cranial defects, which induced rapid initiation of bone regeneration. We co-cultured M0, M1 and M2 macrophage exosomes with BMSCs in a previous study. The results revealed that M1 macrophage exosomes dramatically promoted the BMSC osteogenic genes expression and exhibited markedly enhanced ALP staining and alizarin red staining [26]. In the present investigation, we found that exosomes secreted by both M1 and M2 macrophage promoted MC3T3-E1 osteogenic differentiation, but the effect of exosomes derived from M1 macrophage was more pronounced. This may be related to the fact that M1 macrophage was mainly involved in the recruitment and differentiation of early osteoblasts. In the current work, we have revealed the mechanism by which M1 macrophage can promote MC3T3-E1, and we will continue to explore the effects of M2 macrophage exosomes in bone defect repair in the following studies.

Exosomes secreted by macrophages can inherit the function of mother cells and regulate the immune response by delivering miRNA to target cells [40]. High-throughput sequencing could provide a comprehensive, accurate and efficient screening of specific miRNA information in exosomes [41], hence miRNA sequencing was used in our previous research to examine the miRNA expression profiles of M1 and M2 macrophage exosomes. Sequencing analysis of M1 exosomes revealed that miR-21a-5p was significantly highly expressed [26]. The corresponding miRNAs in the secreted exosomes of primary cells appear to be similarly altered when the expression levels of miRNAs in the cells were altered. The biological activity of the recipient cell will be altered by the transfer of exosomal miRNA when the exosomes are endocytosed by the recipient cell [17]. We constructed macrophage exosomes knocking down and overexpressing miR-21a-5p by transfection technique and transported miR-21a-5p into osteoblasts via exosomes. The results suggested that miR-21a-5p increased osteoblast differentiation of BMSCs [26]. Within the current work, we proved that overexpression of M1 macrophage exosome miR-21a-5p promoted osteogenic gene expression and osteogenic derivation of MC3T3-E1. In contrast, the knockdown of miR-21a-5p inhibited osteogenesis derivation (Figure 2). It has also been reported that small extracellular vesicles from hypoxic mesenchymal stem cells promoted vascularized bone regeneration via the miR-210-3p/EFNA3/PI3K pathway [42].

The rat cranial critical size defect model is a classic model for evaluating the effect of bone repair in bone tissue engineering [43]. Hydrogels show great promise in bone tissue engineering due to their distinctive benefits, namely good biocompatibility and biodegradability, modifiable mechanical properties, excellent scalability and the ability to fill irregular defects by injection [44]. In the current study, we treated rats with cranial defects by hydrogel-loaded M1 macrophage exosomes and M1 macrophage exosomes overexpressing miR-21a-5p, respectively. Bone healing was assessed by CBCT, HE staining and Masson staining. The results demonstrated that M1 macrophage exosome miR-21a-5p promoted bone defect repair *in vivo* (Figures 3–6).

**Figure 10.**
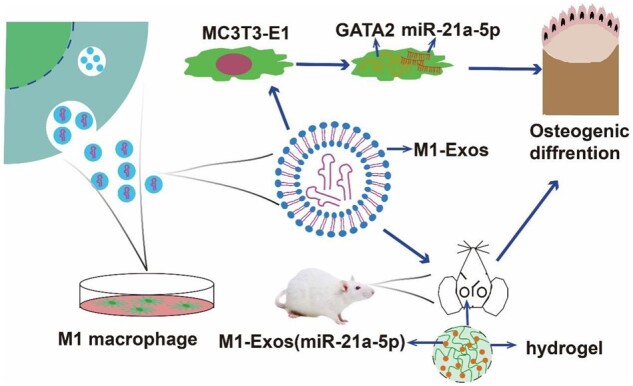
Schematic diagram of M1 macrophage-derived exosome miR-21a-5p targeted GATA2 to promote MC3T3-E1 osteogenic differentiation and bone regeneration.

Next, we focused on the influence of M1 macrophage exosome miRNA-21a-5p on osteogenic differentiation *in vitro* and the mechanism. MiRNAs are endogenous non-coding small RNAs that could regulate at least 30% of the body’s protein gene coding and have low immunogenicity [45]. We overexpressed miR-21a-5p in MC3T3-E1 and found that miR-21a-5p directly promoted MC3T3-E1 osteogenic differentiation (Figure 7). MicroRNAs can restrain target gene transcription and mRNA degradation by binding to the target gene [46]. We identified GATA2 as a key target for miR-21a-5p-mediated osteoblast differentiation of MC3T3-E1. In recent years, it has been found that GATA2 was required for the generation of osteoblastic progenitor cells and that GATA2 could co-regulate the differentiation of osteoblastic precursor cells to osteoclasts with NFATc1 [47]. For validating the modulation regime of miR-21a-5p and GATA2 in MC3T3-E1 cells, we performed a dual luciferase reporter assay and further designed a rescue assay to observe the osteogenic differentiation of MC3T3-E1. Our investigation suggested that miR-21a-5p bound the 3′UTR of GATA2 to downregulate GATA2 expression to reverse the inhibitory effect of GATA2 on osteoblasts, thereby promoting bone regeneration (Figures 8 and 9). Schematic diagram is shown in Figure 10.

## Conclusion

In conclusion, this work gave great support that the M1 macrophage exosome miR-21a-5p promoted bone healing by inhibiting GATA2. Therefore, local injection of M1 macrophage secretion that overexpresses miR-21a-5p was probably a hopeful strategy of treatment to enhance bone repair. Giving the bone immunomodulatory ability to biomaterials by loading them with biological factors will be one of the commonly used methods. Therefore, biomaterials that can load the secretion of macrophages’ exosome can be further developed.

## Data Availability

The full data of the research are contained in this article.
